# Spectroelectrochemical insights into the intrinsic nature of lead halide perovskites

**DOI:** 10.1186/s40580-024-00459-w

**Published:** 2024-11-30

**Authors:** Seonhong Min, Minwook Jeon, Junsang Cho, Jin Ho Bang, Prashant V. Kamat

**Affiliations:** 1 School of Chemistry and Energy, Sungshin Women’s University, Seoul, 01133 Republic of Korea; 2 Department of Applied Chemistry, Center for Bionano Intelligence Education and Research, Hanyang University ERICA, Ansan, Gyeonggi-do 15588 Republic of Korea; 3 Department of Chemical and Molecular Engineering, Hanyang University ERICA, Ansan, Gyeonggi-do 15588 Republic of Korea; 4 Radiation Laboratory, Department of Chemistry and Biochemistry, University of Notre Dame, Notre Dame, IN 46556 USA

**Keywords:** Halide perovskites, Photoelectrochemistry, Spectroelectrochemistry, Instability, Iodide oxidation, Halide migration

## Abstract

Lead halide perovskites have emerged as a new class of semiconductor materials with exceptional optoelectronic properties, sparking significant research interest in photovoltaics and light-emitting diodes. However, achieving long-term operational stability remains a critical hurdle. The soft, ionic nature of the halide perovskite lattice renders them vulnerable to various instabilities. These instabilities can be triggered by factors such as photoexcitation, electrical bias, and the surrounding electrolyte/solvent or atmosphere under operating conditions. Spectroelectrochemistry offers a powerful approach to bridge the gap between electrochemistry and photochemistry (or spectroscopy), by providing a comprehensive understanding of the band structure and excited-state dynamics of halide perovskites. This review summarizes recent advances that highlight the fundamental principles, the electronic band structure of halide perovskite materials, and the photoelectrochemical phenomena observed upon photo- and electro-chemical charge injections. Further, we discuss halide instability, encompassing halide oxidation, vacancy formation, ion migration, degradation, and sequential expulsion under electrical bias. Spectroelectrochemical studies that provide a deeper understanding of interfacial processes and halide mobility can pave the way for the design of more robust perovskites, accelerating future research and development efforts.

**Graphical Abstract:**

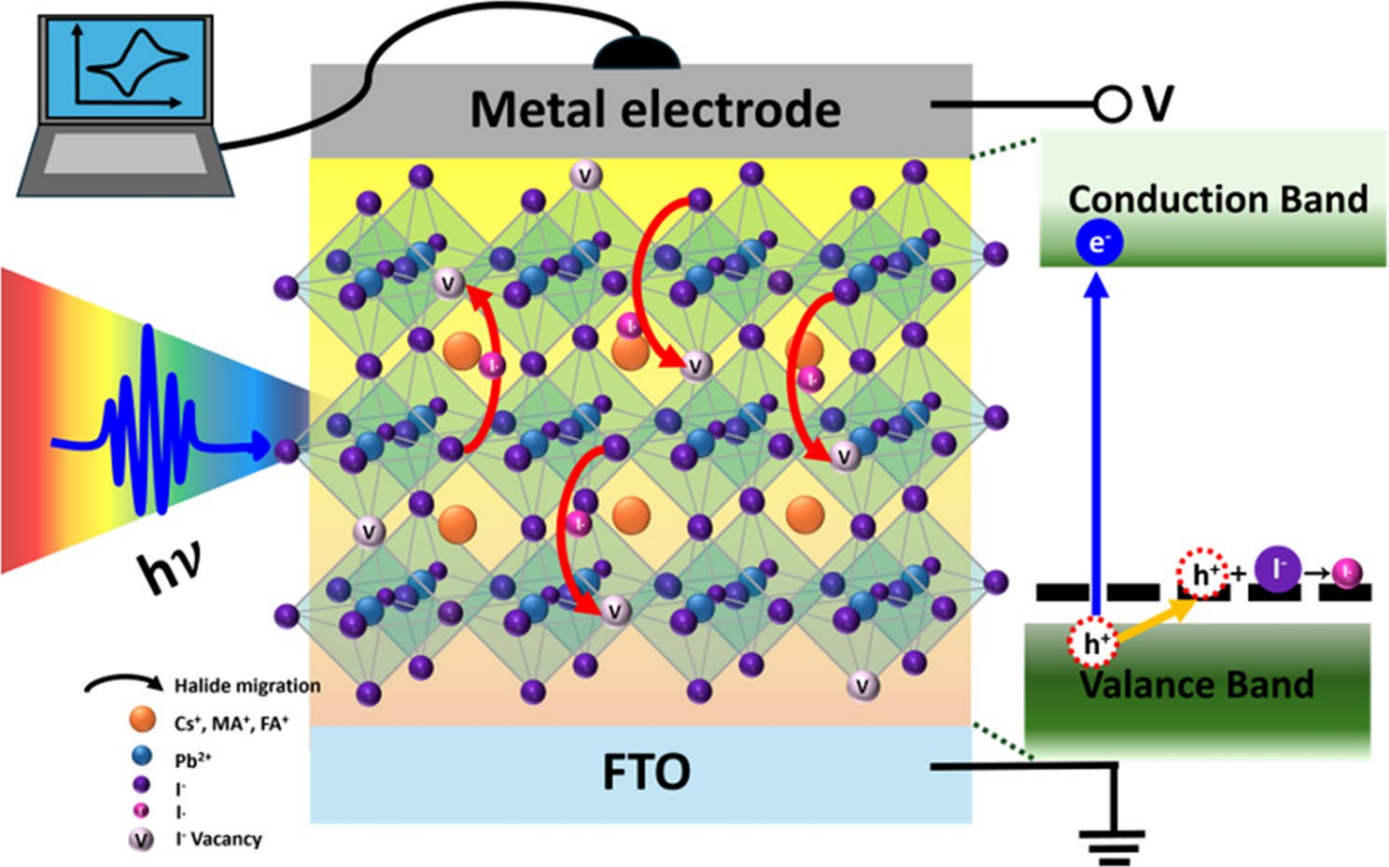

## Introduction

Lead halide perovskites have emerged as next-generation optoelectronic materials due to their exceptional properties. These properties include a high absorption coefficient, near-unity photoluminescence (PL) quantum yield, long carrier lifetime, and tolerance to defects [1–8]. Coupled with their solution processability, these advantages make perovskites highly attractive candidates for photovoltaics (PVs), light-emitting diodes (LEDs), photodetectors, and lasers [9–13]. Notably, perovskite solar cells (PSCs) have achieved impressive power conversion efficiencies exceeding 26%, and green-emitting perovskite LEDs with external quantum efficiencies surpassing 28.9%. However, a significant hurdle to their practical implementation and commercialization remains as they are susceptible to ambient operation conditions (oxygen and moisture), light exposure, heat, and electric field [6, 14–18].

The soft, ionic lattice of lead halide perovskites, often referred to as liquid crystalline, arises from the presence of highly electronegative halide ions [19, 20]. This property is in stark contrast to the strong covalent bonding found in traditional semiconductor solids (e.g., CdSe and InAs) [3, 21, 22]. The ionic nature of these materials facilitates halide migration throughout the crystal lattice. This allows for post-synthetic halide ion exchange even under ambient conditions when exposed to other halide sources [23–29]. Furthermore, the ionic bonding contributes to the instability of mixed halide perovskites, leading to halide segregation under an electric field or photoexcitation [30–34]. This soft, ionic lattice structure acts as a double-edged sword. While it allows facile tuning of bandgap through varying halide composition, it also introduces instability arising from mobile halide ions. Under external stimuli such as light (photons) and electrical fields (electron or hole), these semiconducting materials become even more susceptible to halide migration (defect transport) and subsequent degradation [35–39]. Therefore, understanding the mechanisms behind the instability of halide perovskites is crucial for addressing their long-term operational stability in optoelectronic devices.

Spectroelectrochemistry (including photoelectrochemistry) has emerged as a useful tool for investigating light-induced processes in perovskite film as well as interfacial processes under applied electrochemical bias. Using this in-situ spectroscopic tool, one can probe the charge transfer events under external photochemical and electrochemical charge injection [40–48]. Although halide perovskites show instability in polar solvents, one can overcome this challenge by careful selection of electrolytes and solvents. Samu et al. established optimal electrolyte concentrations (0.01‒0.1 M) and solvent types for stable spectroelectrochemical measurements of halide perovskites, paving the way to conduct electrochemical experiments reliably [44, 49, 50]. DuBose et al. further utilized a combination of steady-state and transient absorption spectroscopy in conjunction with electrochemistry to elucidate the halide segregation mechanism and stability of mixed halide perovskites [51–53]. Their work revealed how photochemical and electrochemical hole injections can induce iodide oxidation that in turn leads to halide ion segregation (Br/I) followed by iodide expulsion. Spectroelectrochemistry also allows for in-situ visualization of these changes, such as band edge peak shifts or attenuation, offering valuable insights into material behavior under charge injection conditions [49, 51, 54]. Despite the mechanistic information obtained from these techniques, research efforts in photoelectrochemical and spectroelectrochemical measurements of halide perovskite films remain limited.

A recent surge in research activity highlights the promising potential of halide perovskites for photocatalytic, electrocatalytic, and photoelectrochemical applications [55–60]. However, their operational stability under light and electrical stimulation remains a critical challenge. In this review, we discuss recent findings emerging from photo- and spectroelectrochemical studies. These techniques, which couple electrochemical and photochemical (or spectroscopy) measurements, offer a comprehensive understanding of key factors influencing the performance and stability of the halide perovskites, which include charge injection (photonic or electronic), charge carrier recombination and transport at interfaces, and excited-state properties. These studies also explore how halide instability arises from halide ion oxidation followed by migration, leading to both reversible and irreversible changes depending on the applied potentials from the thermodynamic and kinetic perspectives. The mechanistic insights gained from these studies further contribute to the understanding of halide perovskite stability, encompassing migration and oxidation of halide ions, doping, and degradation.

## Operational stability of lead halide perovskites

### Photochemical stability of halide perovskites

Given the promises of halide perovskites in various applications, it is important to address their instability issues [61]. Although perovskite-based devices exhibit superior performance in laboratory settings, their poor stability under ambient conditions, in oxygen and water [62], light [63], and electric stress [64] substantially hampers their practical applications.

**Fig. 1:**
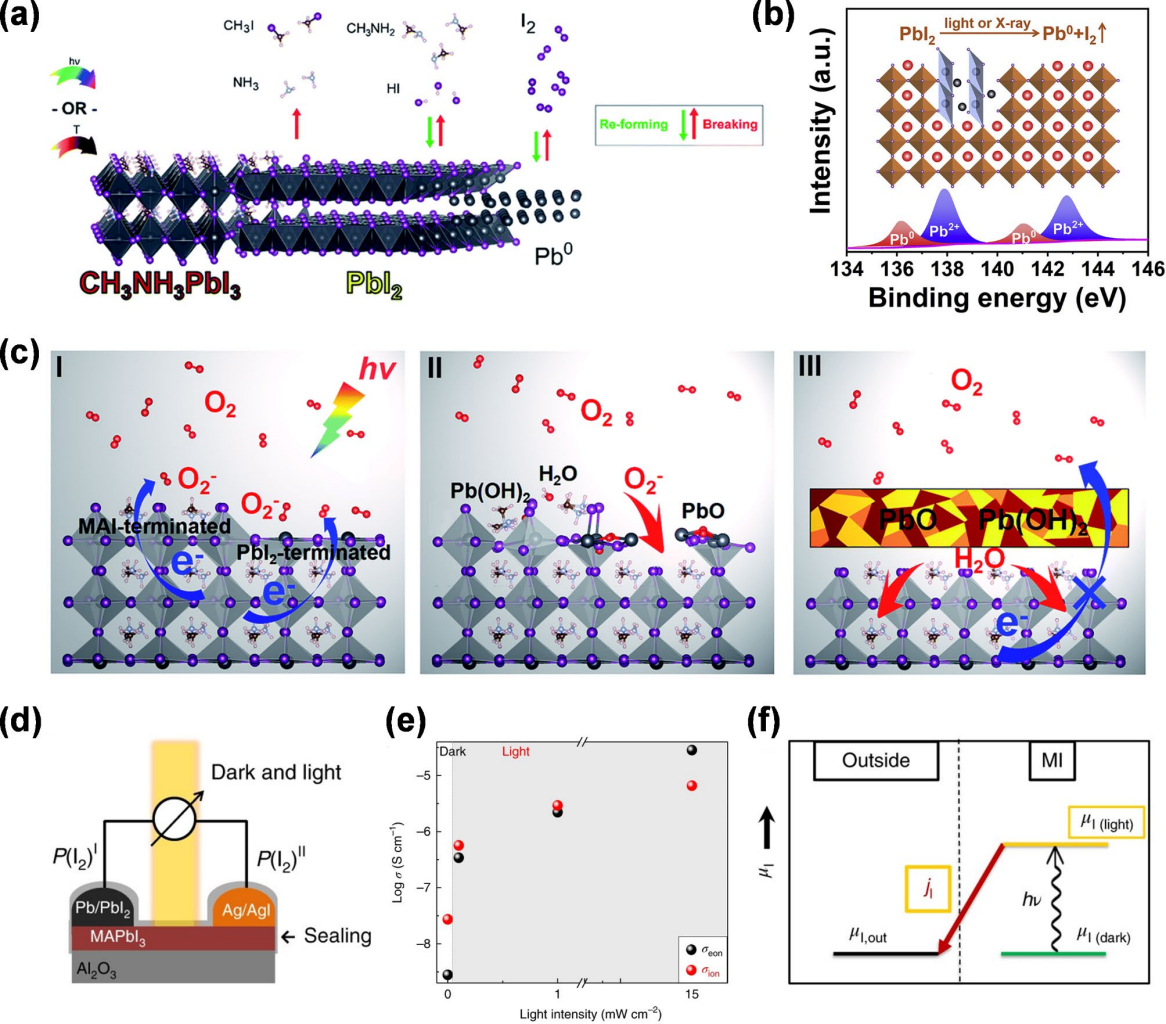
(**a**) MAPbI_3_ photodecomposition and thermal degradation processes leading to irreversible decomposition to organic volatile gas species (CH_3_I + NH_3_), reversible decomposition (CH_3_NH_2_ + HI), and reversible generation of I_2_ and nonvolatile Pb^0^ under illumination or mild heat conditions. Reproduced with permission [66]. Copyright 2018, Royal Society of Chemistry. (**b**) Decomposition of MAPbI_3_ under light or X-ray exposure leading to the formation of Pb^0^ and I_2_, associated with iodine vacancies. Reproduced with permission [67]. Copyright 2022, Elsevier. (**c**) Schematic illustration of the photo-oxidative degradation process of the MAPbI_3_. Reproduced with permission [68]. Copyright 2019, Royal Society of Chemistry. (**d**) Open-circuit voltage battery cell wherein MAPI acts as the solid electrolyte. (**e**) DC galvanostatic polarization experiment performed at 40 °C under an Ar atmosphere to decouple ionic (σ_ion_) and electronic (σ_eon_) contributions. (**f**) Change in the chemical potential diagram of iodine over the equilibrium value under light illumination (MI stands for metal iodide and outside refers to zero iodine concentration). (**d-f**) Reproduced with permission [75]. Copyright 2018, Nature Publishing Group

Under photoirradiation, bandgap excitation of halide perovskites induces ion migration. The halide ion migration leading to structural and functional disruptions remains one of the major factors responsible for their degradation [65]. For example, the creation of iodine vacancies from the loss of volatile MAPbI_3_ (CH_3_NH_3_I) leads to an increase in trap states within the perovskite materials. During the degradation process, the formation of Pb^0^ through photoreduction has also been observed following photoexcitation as a main decomposition product of MAPbI_3_. This critically impairs the performance of PSCs (Fig. 1a) [66]. The defect centers represented by Pb^0^ inhibit effective charge transfer and increase non-radiative recombination, subsequently reducing the performance of the cells (Fig. 1b) [67]. When exposed to ambient conditions, viz., in the presence of oxygen and moisture, halide perovskites can undergo further degradation [42]. This degradation process is initiated with the formation of superoxide ions (O^2‒^) which rapidly react with photo-generated carriers to accelerate degradation. The presence of O^2‒^ is particularly problematic as they react with MA^+^ and Pb^2+^ ions in MAPbI_3_ to form Pb(OH)_2_ and H_2_O. The resultant Pb(OH)_2_ further oxidizes to create PbO, which forms a protective layer on the perovskite surface that somewhat mitigates further oxidation. However, the water molecules produced during this reaction can induce additional degradation (Fig. 1c) [68]. Moreover, light exposure significantly reduces the migration energy barrier for ions, such as MA^+^, H^+^, and I^‒^, within the perovskite [69]. This decrease is attributed to the formation of randomly distributed ion vacancies within the crystal structure. Such ion migration not only weakens the material but also alters its electronic properties. Following light exposure, these ion vacancies migrate to the interface between the hole transport layer (HTL) and the perovskite layer, where they form a Debye layer. This layer acts as a barrier to charge extraction, further diminishing the overall performance of the device [70].

### Photoelectrochemical stability of halide perovskites

The photoelectrochemical stability of perovskites is a critical area of focus when considering the operational longevity and efficiency of these materials in various applications. Under device operation conditions including light (e.g., solar cell) or electric field (e.g., LED), halide perovskites often exhibit degradation due to the ease of halide migration. Perovskite films exhibit both ionic and electronic conductivities [71–73]. To decouple these two contributions to overall conductivities, a battery cell comprised of MAPbI_3_ as a solid electrolyte was devised to carry out DC galvanostatic polarization measurements under dark and light excitation (Fig. 1d). Even at light intensity of 1 mW cm^‒2^, an increase in ionic contribution by two orders of magnitude was observed. While the increase in electronic conductivity under light illumination was explained by an increase in charge carrier concentration upon bandgap excitation, the increase in ionic contribution (σ_eon_) was unexpected (Fig. 1e). The rationale behind significant increased ionic conductivity is closely associated with photo-induced halide ion migration. Here, the nature of ionic conduction is associated with the iodine vacancy, facilitating halide ion migration. During bandgap excitation, iodide oxidation and the formation of neutral iodine species migrate into interstitials, generating additional iodide ion vacancy. Thus, initial iodide oxidation (and its oxidation potential) and its migration into interstitial sites result in increased ionic conductivity. Iodine flux within the crystal lattice can ultimately lead to its expulsion, exceeding the equilibrium homogeneity range of the material (Fig. 1f). This is exemplified by the irreversible degradation of MAPbI_3_ when subjected to electric field stress. The decomposition resulting in yellow PbI_2_ and MAI starts near the anode and then progresses throughout the film [74].

The reversibility of the transformation is determined by the oxidized neutral iodine: whether it is in the crystal lattices (reversible) or removed from the lattices (irreversible). If iodine is removed from the lattice irreversibly forming an iodine gradient across the halide perovskite lattices (across the film), it can change the thermodynamic equilibrium of iodine species (Fig. 1f). Light-induced iodine flux (iodine non-stoichiometry) can serve as a thermodynamic (chemical potential) driving forces to induce halide ion migration (kinetics), which ultimately lead to decomposition through iodine expulsion into solvent or vacuum [38, 75]. Moreover, ion migration significantly affects hysteresis responses during PV measurements, posing challenges to photoelectrochemical stability. In the initial dark state, the system is in equilibrium with no significant changes. As the illumination time extends, carrier accumulation at the interface is induced, leading to the formation of electrostatic potential. This increase in potential contributes to band bending within the semiconductor, altering the concentration and distribution of charge carriers [76]. Such changes increase recombination rates at the interface, subsequently impacting the electrical performance of the device.

The quantification of ionic diffusion in perovskites has been demonstrated through impedance spectroscopy [77]. It is highlighted that increased crystal thickness enhances ion transit time to external interfaces, as evidenced by the impedance response, where a loop in the low-frequency region signifies inductive behavior and negative capacitance. A distinctive negative capacitance characteristic associated with the occurrence of inverted hysteresis has been elucidated, providing insights into additional recombination mechanisms directly linked to degradation processes [78]. By employing the surface polarization model, a time constant associated with the kinetics of ion/vacancy interactions at the surface was obtained, suggesting the presence of additional recombination paths at the interface between the TiO_2_ and perovskite layers. This change in recombination dynamics was attributed to the accelerated degradation and hysteresis behavior of PSCs. Thus, electrochemical investigations are pivotal in elucidating the complex relationship between halide ion migration and the causes of performance degradation. To enhance the structural and chemical robustness and photoelectrochemical stability of perovskites, it is essential to clearly understand the fundamental mechanisms of halide ion migration occurring under various environmental conditions along with conducting thorough assessments of both structural and chemical stability over prolonged operational cycles. Crucially, spectroelectrochemical analysis emerges as an indispensable tool in this research endeavor, enabling precise determination of the requisite voltage ranges and material stability for catalytic applications within photoelectrochemical systems.

## Fundamentals of lead halide perovskite spectroelectrochemistry

### Bandgap estimation of lead halide perovskites

**Fig. 2:**
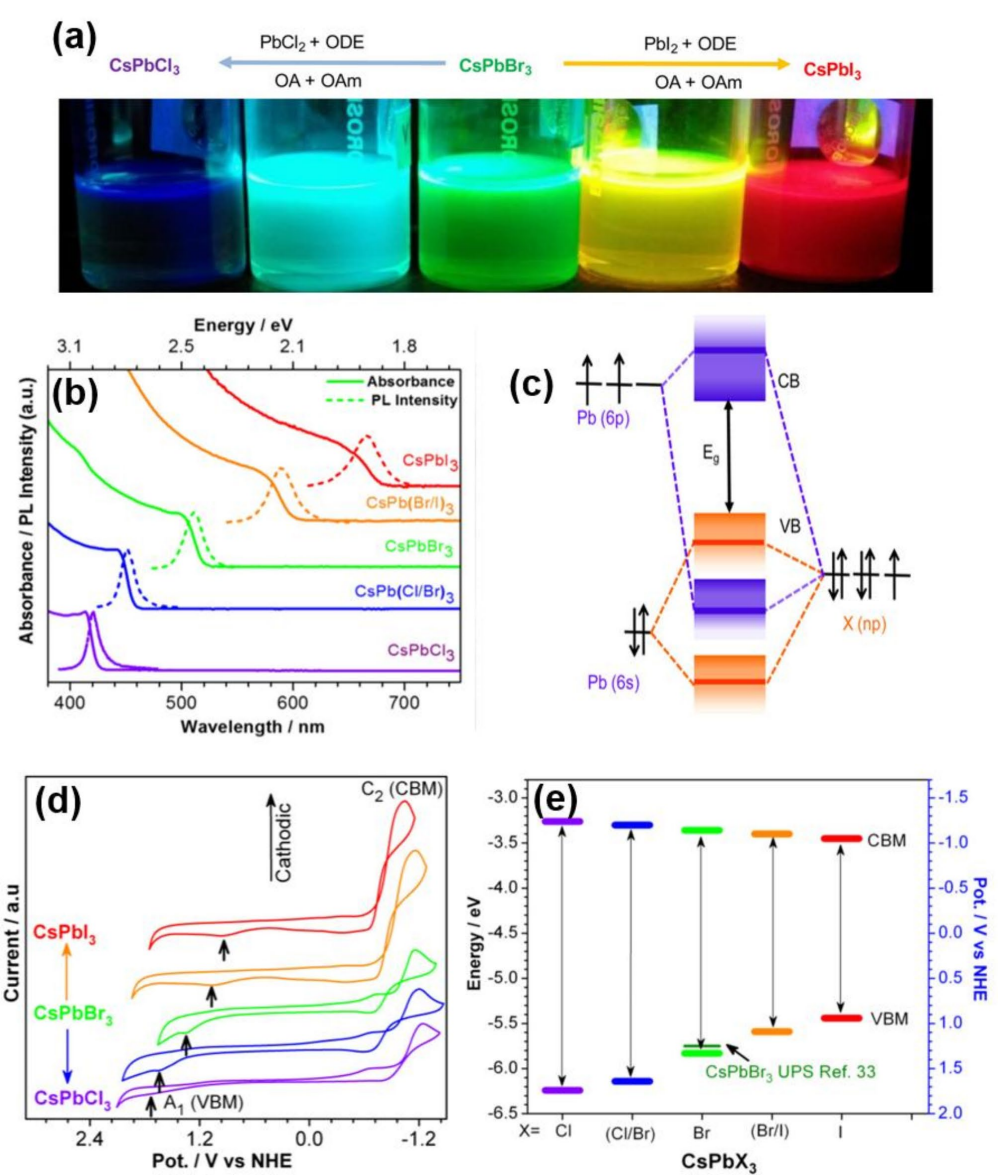
(**a**) Digital photograph of colloidal dispersion of CsPbX_3_ (X = Cl, Br, or I) halide perovskite nanocrystals in a solution taken under UV lamp excitation (365 nm). (**b**) Corresponding UV-vis absorption and PL emission spectra of CsPbX_3_ nanocrystals as a function of varying halide composition. (**c**) Electronic band structure of halide perovskites. (**d**) Cyclic voltammograms of CsPbX_3_ nanocrystals dispersed in 50 mM TBAP solution as a function of halide composition. (**e**) Corresponding valence and conduction band edge potential determined by CV and UV-vis absorption (spectroelectrochemistry). (**a-e**) Reproduced with permission [2]. Copyright 2016, American Chemical Society

Understanding the band energies of lead halide perovskites is important for designing optoelectronic devices and achieving greater stability. An interesting property of lead halide perovskites is its bandgap tunability achieved through varying halide composition in APbX_3_ (A = Cs, MA (methylammonium), or FA (formamidinium); X = Cl, Br, or I) crystal structures [79, 80]. Figure 2a presents the characteristic PL emission colors of lead halide perovskite nanocrystal dispersions in solution as a function of the halide type under UV excitation (365 nm). These spectral profiles show the tunable PL properties of CsPbX_3_ (X = Cl, Br, or I) and their mixed halide counterparts (Cl/Br or Br/I). As the halide constituent transitioned from Cl to Br and then to I, the bandgap decreased from 2.9 to 1.8 eV, which corresponded to a red-shift in the PL emission wavelength, ranging from 410 to 700 nm (Fig. 2b).

The halide *n*p orbitals mainly contribute to the valance band edge whereas the energy of Pb 6p orbitals determine the conduction band edge, as illustrated in the molecular orbital diagram in Fig. 2c. The molecular orbital diagram shows that the valence band edge is the antibonding hybridization mainly comprised of Pb 6s and X *n*p orbitals, with major contribution from X *n*p. With moving from I (5p) to Br (4p) and Cl (3p), the energy level of the halide *n*p orbital decreases, shifting the valence band maximum toward more negative (deeper) potentials. The valence band edge potential is dictated by the energy of the halide *np* orbitals (Cl 3p, Br 4p, and I 5p), thus making them dependent on the nature of the halide component (Fig. 2e) [40, 81–84]. These band alignments are measured using an electrochemistry setup in which electrons or holes are injected through applied potential. Cyclic voltammetry (CV) profiles shown in Fig. 2d exhibit increased current near the band edges as one sweeps the potential across the anodic and cathodic regions. CV measurements utilize nanocrystal dispersions or perovskite films in the presence of an electrolyte solution. Common solvents for this purpose include toluene or dichloromethane (DCM) or a mixture of the two. However, caution should be exercised while conducting electrochemical measurements since the limited polarity of these solvents can render internal resistance or *IR* drop difficult to fully compensate. In addition, the instability of the perovskite nanocrystals during prolonged exposure to a solvent medium may also hinder accurate electrochemical measurements.

### Spectroelectrochemistry of lead halide perovskites: insight into photoelectrochemical stability

**Fig. 3:**
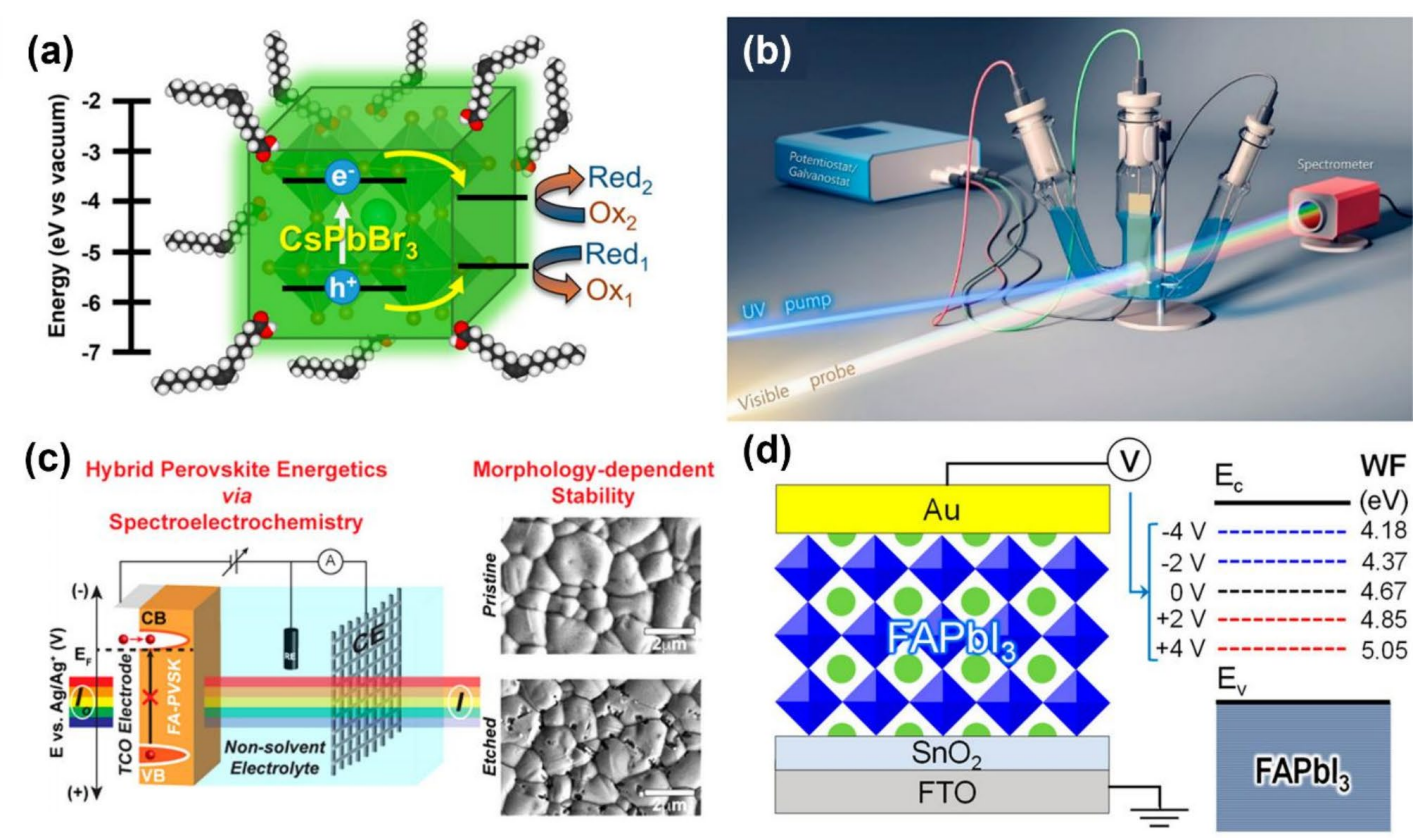
(**a**) Schematic illustration of charge carrier dynamics and photocatalytic redox reaction process occurring at the surface of perovskite nanocrystals upon bandgap excitation. Reproduced with permission [87]. Copyright 2022, American Chemical Society. (**b**) Spectroelectrochemistry setup with three electrodes. Reproduced with permission [44]. Copyright 2018, American Chemical Society. (**c**) Schematic illustration of the study of halide perovskites using spectroelectrochemistry and SEM measurements to elucidate the stability given potential. Reproduced with permission [45]. Copyright 2017, American Chemical Society. (**d**) Schematic illustration of work function changes in a perovskite-based device (FTO/SnO_2_/FAPbI_3_/Au) with applied different voltage bias. Reproduced with permission [85]. Copyright 2021, American Chemical Society

Perovskite-based devices rely on the generation of charge carriers for their operation. The photochemical approach utilizes photons to achieve charge separation and following a photochemical reaction (Fig. 3a), while the electrochemical approach selectively injects electrons or holes into semiconductors. These two external stimuli that activate perovskite semiconductors are convenient to initiate photo- and electrochemical reactions. Spectroelectrochemistry combines electrochemical methods with spectroscopic measurements (UV-vis absorption, PL, Infrared, Raman, or time-resolved spectroscopy) to understand the complex interplay between photochemical and electrochemical reactions under operational conditions. Monitoring spectroscopic changes in the semiconductor during electrochemical stimulation (Fig. 3b, typically using a three-electrode system with working, counter, and reference electrodes) allows spectroelectrochemical experiments to gather detailed information on the electronic band structure modifications. The ability to distinguish the response to selective charge injection (electrons or holes) through their spectroscopic signatures makes this technique a valuable resource [45]. A representative example that utilizes spectroelectrochemistry to study the stability of lead halide perovskites under specific electrochemical conditions is shown in Fig. 3c. This approach when combined with structural characterizations using X-ray diffraction and scanning electron microscopy (SEM) can aid in gaining a comprehensive understanding of the stability of halide perovskites. Such an interplay between the spectroscopic changes and the structural morphology modifications as a function of the applied electrochemical bias is useful to gain additional mechanistic insights. The ability to tune the work function of lead halide perovskites using external electric bias, without introducing compositional changes or surface modifications enabled the modulation of the energy difference between the electrode’s Fermi level and the perovskite’s work function [85, 86]. For example, the applied bias can induce n- or p-type charge transfer doping as it controls the Fermi level (Fig. 3d) [85].

### Boundary conditions for spectroelectrochemistry of lead halide perovskites

**Fig. 4:**
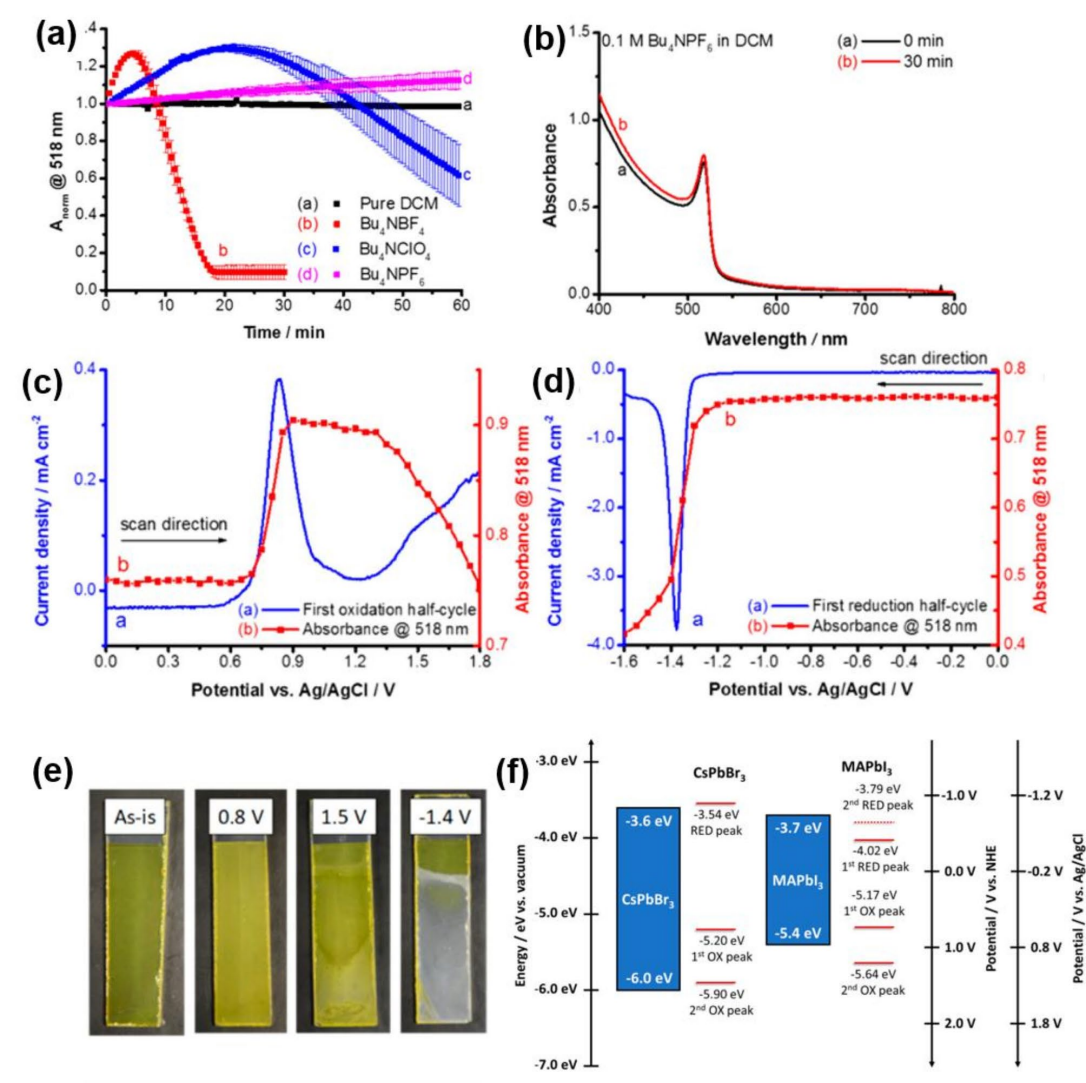
(**a**) Normalized absorbance changes at band-edge absorption of CsPbBr_3_ deposited on TiO_2_/FTO electrode in DCM containing different salts. (**b**) Absorption changes before (0 min) and after (30 min) immersion in DCM with 0.1 M TBABF_4_. Absorbance and current density changes of CsPbBr_3_/TiO_2_/FTO electrode during (**c**) the oxidation cycle and (**d**) the reduction cycle. (**e**) Digital photographs of an electrode at a given potential. The sweep rate is 10 mV s^‒1^ in 0.1 M TBABF_4_/DCM. (**f**) Band position and redox potential of CsPbBr_3_ and MAPbI_3_ relative to the normal hydrogen electrode (NHE) or Ag/AgCl. (**a-f**) Reproduced with permission [44]. Copyright 2018, American Chemical Society

Careful selection of the solvent and choice of electrolyte allows reliable electrochemical measurements within a specific time window in which the perovskite film remains stable (as shown in Fig. 3c) [44, 50, 88]. Utilizing an electrolyte that provides stability of halide perovskites under operative conditions is an important consideration for gathering information regarding their electronic structures and electrochemical response during electron and hole injection. For example, by monitoring absorbance changes in a CsPbBr_3_ film deposited on a TiO_2_/fluorine-doped tin oxide (FTO) electrode, it is possible to screen suitable solvent and electrolyte composition for spectroelectrochemical measurements (Fig. 4a). This approach has been used to establish the stability of CsPbBr_3_ films in DCM solvent with tetrabutylammonium hexafluorophosphate (TBAFP_6_) as the supporting electrolyte. The minimal changes in the absorbance of CsPbBr_3_ film before and after exposure to the electrolyte containing TBAPF_6_ confirmed the stability of the perovskite film in DCM for 30 min (Fig. 4b). As anticipated, considerations regarding the solvent type, electrolyte choice, and halide perovskite film stability are unnecessary when utilizing all-solid-state perovskite cells, as solid-state charge conducting/transport layers are employed instead. By sweeping electrochemical potentials in the positive and negative region of the electrochemical window and simultaneously recording absorbance and current density changes, it was possible to establish the stability window for carrying out electrochemical experiments (Fig. 4c‒e).

The band-edge energies of halide perovskites, which are governed by their electronic band structures, determine a stable potential window for electrochemical measurements (Fig. 4f). The oxidation and reduction cycles revealed that CsPbBr_3_ is stable in the electrochemical window of ‒1.0 ~ + 0.6 V. The increase in current during the positive (anodic) and negative (cathodic) scans from 0 V to each bias potential with a distinctive peak position was attributed to the valence and conduction band-edge potential, respectively. The increase in current was associated with interfacial charge transfer between halide perovskite and electrode (Fig. 4c, d). As compared to bromide perovskite, iodide perovskite film was more susceptible to degradation in DCM containing 0.01 M TBAPF_6_ as seen from the stability window of ‒0.65 ~ + 0.55 V. Beyond the stable electrochemical window, the perovskite film underwent an irreversible electrochemical reaction that occurred as shown in Fig. 4e (which will be further discussed in detail later).

### Electrochemical charge injection into halide perovskites: modulation of charge carrier dynamics with bias

**Fig. 5:**
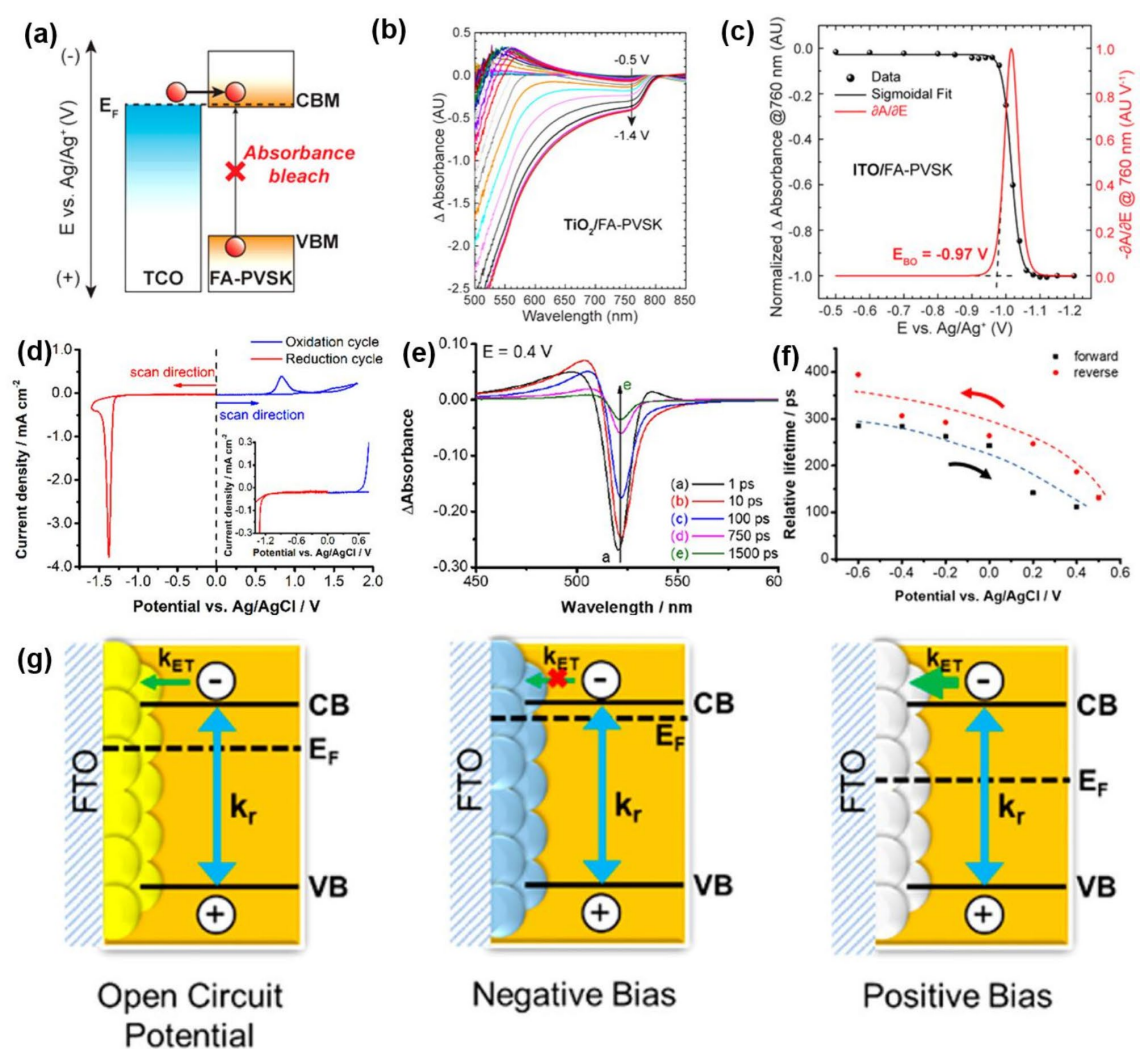
(**a**) Electron injection under negative potential when Fermi energy level of electrode reaches the conduction band of perovskites featured with distinctive absorbance bleach from optical absorbance shown in difference absorbance spectra given bias. (**b**) Difference absorption measurement of FAPbI_3_ perovskites film deposited on ITO substrates (ITO/FAPbI_3_) electrode immersed in a 0.1 M TBAPF_6_ in DCM with applied electric field. (**c**) Normalized absorbance bleach peak at 760 nm of iodide perovskite films (ITO/FAPbI_3_) as a function of applied potentials. (**a-c**) Reproduced with permission [45]. Copyright 2017, American Chemical Society. (**d**) CV measurements of FTO/TiO_2_/CsPbBr_3_ films in DCM with 0.1 M TBAPF_6_ electrolyte. (**e**) Transient absorption spectra of recorded FTO/TiO_2_/CsPbBr_3_ films upon 387 nm laser pulse excitation in a spectroelectrochemical cell containing 0.1 M TBAPF_6_ electrolyte in DCM at an applied potential of + 0.4 V. (**f**) Modulation of bleach recovery (or charge carrier recombination) lifetime as a function of applied potentials (V vs. Ag/AgCl). (**g**) Schematic demonstration of charge carrier dependence as a function of applied potentials, showing a change in Fermi energy level of electrode and corresponding rate constant of charge carrier recombination rate (*k*_r_) with open circuit, negative bias, and positive bias, respectively. (**d-g**) Reproduced with permission [88]. Copyright 2018, American Chemical Society

Spectroelectrochemical experiments can be further extended to probe the effects of charge injection (electrons or holes) under controlled electrochemical bias. Figure 5a demonstrates electron injection from the electrode to FAPbI_3_ perovskites (a bandgap of 1.5 eV) under a negative potential. The Fermi energy level of the electrode varied depending on the applied potential, enabling electron or hole injection into the halide perovskites [52, 88, 89]. Steady-state absorption measurements (including difference absorbance) revealed distinct ground-state bleaching upon applying a sufficient negative bias (Fig. 5b). This bleaching signified electron injection into the halide perovskite, leading to the filling of empty levels in the conduction band. The ground-state bleach feature arose due to the conduction band becoming saturated with electrons. The magnitude of the ground-state bleach feature intensified as the excited states became saturated with charge carriers at more negative potentials (Fig. 5b). A sigmoidal fit of the negative bleach (Fig. 5c) represents the saturation of the bleach features at a given negative potential through conduction band charging. The first derivative of the absorbance bleaching (dA/dE) is proportional to the Faradaic current density, signifying its correspondence to the voltammetric current response under the electrochemical bias. The first bleach onset potential, determined by linear extrapolation of the dA/dE plot, corresponds to the conduction band edge potential with reference to a reference electrode (Ag/Ag^+^ or NHE) (Fig. 5a, c).

Ground-state bleaching phenomenon arising from band filling of photogenerated electrons was also observed in time-resolved transient absorption measurements under applied potential. Transient absorption measurements of CsPbBr_3_ film deposited on an electron transport layer (corresponding CV profile depicted in Fig. 5d) were performed as a function of pump-probe delay time in the picosecond timescale with application of different electrochemical potentials (Fig. 5e). The applied potential influences electron transfer from perovskite layer into ETL, and thus alters the bleach recovery kinetics. The average charge carrier lifetime decreased as the applied potential was switched to positive potentials. Figure 5f and g illustrate the change in the relative potential of the electrode’s Fermi energy as a function of applied bias and its subsequent effect on charge carrier recombination kinetics. Applying a positive potential lowers the Fermi energy of the electrode, facilitating electron transfer from the halide perovskite to the electron transport layer (TiO_2_/FTO). At negative bias the Fermi Level moves closer to the conduction band, making electron transfer less favorable (Fig. 5g).

### Electrochemical bias to modulate Fermi level in halide perovskites

**Fig. 6:**
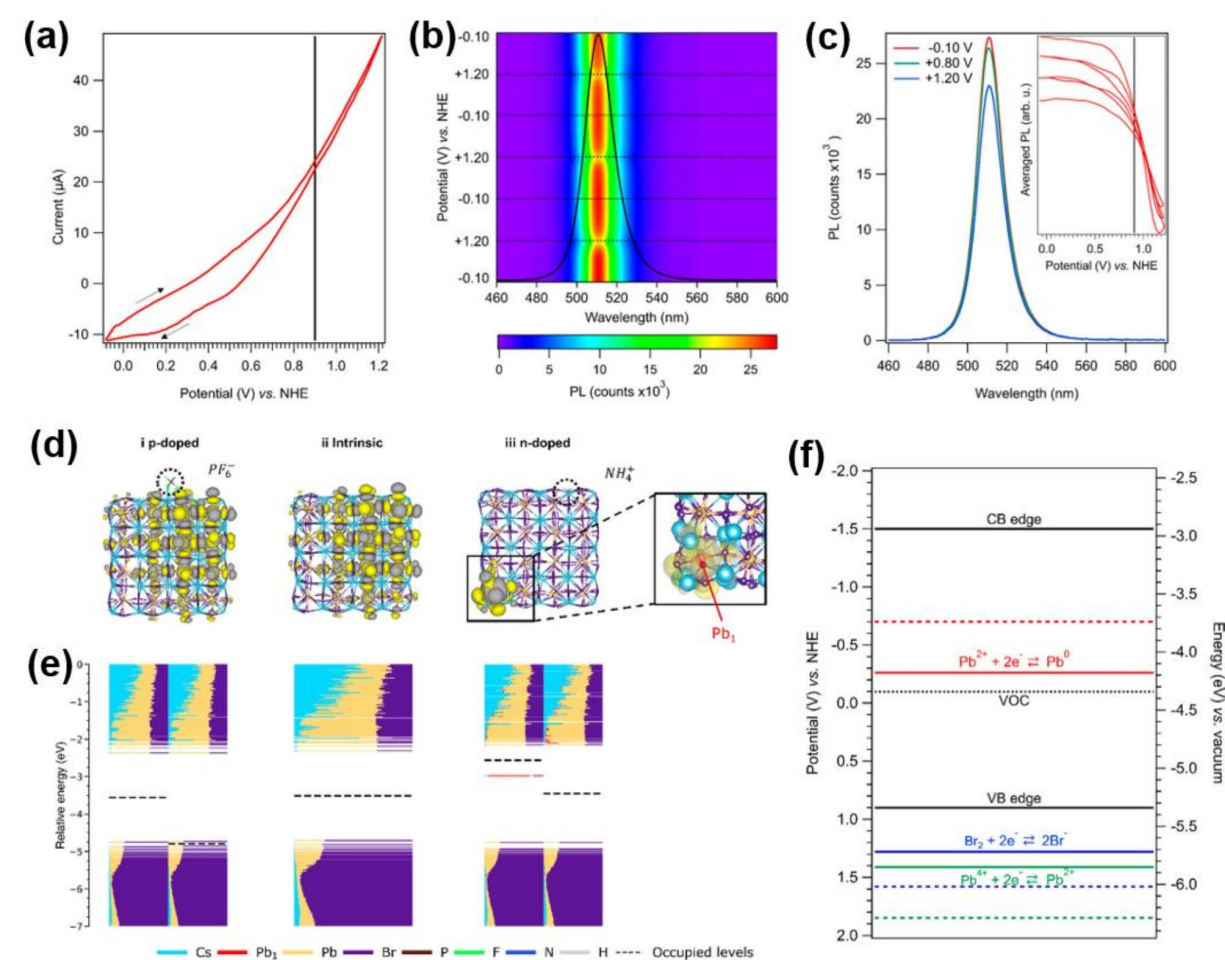
(**a**) Cyclic voltammogram of CsPbBr_3_ nanocrystals electrode measured at a scan speed of 20 mV s^‒1^. (**b**) Two-dimensional (2D) PL emission spectra color mapping at applied potential from ‒0.10 V to + 1.20 V. (**c**) PL emission spectra recorded at the given potential of ‒0.10 V, + 0.80 V, and + 1.20 V, respectively. (**d**) DFT calculation of CsPbBr_3_ nanocrystals with contour plots. (**e**) The density of state of each nanocrystal with each atom contribution of Cs, Pb, Br, P, F, N, and H, respectively. (**f**) Corresponding energy diagram of CsPbBr_3_ nanocrystals with valence band (+ 0.9 V), conduction band (‒1.5 V), and open-circuit voltage (‒0.1 V), respectively, along with possible oxidation and reduction reactions. (**a-f**) Reproduced with permission [90]. Copyright 2021, American Chemical Society

Injection of holes or electrons into the semiconductor at controlled applied potentials is a convenient approach to modulate the Fermi energy level. The process of charge accumulation or depletion within the band structure remains reversible as long as the semiconductor is not subjected to anodic or cathodic corrosion. Recent studies employing spectroelectrochemical absorption and PL experiments, combined with density functional theory (DFT) calculations, have demonstrated that reversible p-type behavior in halide perovskites can be achieved through electrochemical hole injection [90]. However, irreversible chemical changes that occur at negative bias as a result of Pb^2+^ reduction limited the study to probe n-type behavior. Figure 6a and b show CV responses (Fig. 6a) and corresponding PL emission color map (Fig. 6b) of CsPbBr_3_ nanocrystal cast on ITO electrode as a function of alternating the potential from ‒0.1 to 1.2 V reversibly. Interestingly, the PL emission was quenched at + 1.2 V and recovered to its initial intensity during the reverse scan. Figure 6c shows the corresponding changes in the PL emission spectra upon applying anodic bias (+ 0.8 V and + 1.1 V) compared to the initial bias (‒0.1 V). DFT calculations suggest that injected holes were primarily delocalized in the valence band, leading to a reversible phenomenon (Fig. 6d, e). In contrast, electron injection resulted in their localization and reduction of Pb²⁺, causing irreversible chemical transformations and degradation of the perovskite. Figure 6f displays the electronic structure along with the redox potentials of chemical species such as Pb²⁺ and Br⁻. The energy diagram indicates that when a negative potential is applied, leading to electron injection, cathodic decomposition readily occurs via the formation of Pb^0^ before electron injection into the conduction band (Eq. 1):

1 $$\text{C}\text{s}\text{P}\text{b}\text{B}\text{r}_3\hspace{0.17em}+\hspace{0.17em}2\text{e}^{-}\:\to\:\:\text{P}\text{b}^{0}\hspace{0.17em}+\hspace{0.17em}\text{C}\text{s}^{+}\:+\:3\text{B}\text{r}^{-}$$

In contrast, anodic bias induces reversible p-doping through hole injection into the valence band. With a more positive potential excess, halide oxidation and degassing (or expulsion from lattice sites) become the primary decomposition pathway (Eq. 2):

2 $$\text{C}\text{s}\text{P}\text{b}\text{B}\text{r}_{3}\:\to\:\:\text{P}\text{b}^{2+}\:+\:\text{C}\text{s}^{+}\:+\:3/2\text{B}\text{r}_{2}\hspace{0.17em}+\hspace{0.17em}3\text{e}^{-}$$

The formal potentials for anodic decomposition are more positive than the CsPbBr_3_ valence band, at 1.3 V (Br^‒^ to Br_2_ oxidation) and 1.4 V (Pb^2+^ to Pb^4+^ oxidation), respectively. Notably, increased anodic bias (exceeding the reversible window) leads to irreversible halide expulsion from perovskite lattices, causing degradation and PL quenching. In specific cases, depending on the surface-passivating ligand and terminal surface of halide perovskites, reversible PL quenching and recovery can be observed. In general, however, anodic bias promotes halide oxidation followed by halide ion migration across the lattices, which will be discussed in detail in a subsequent session [38, 40, 75, 83].

## Reversible and irreversible changes in lead halide perovskites: insight into stability

### Role of iodide oxidation in halide migration

PVs and LEDs are representative perovskites-based optoelectronic devices operating under reverse (former) and forward (latter) bias, respectively [91–95]. In the PV modules, the reverse bias facilitates the separation of electrons and holes to create potential differences in electrodes which leads to current generation in bypass diodes [96–98]. In contrast, forward bias in LED devices promotes the recombination of electrons and holes to produce light emission [99, 100]. Depending on the number of cells and the bypass diodes in the PV and LED modules, applied reverse bias can be increased up to 10 V [94, 97, 99].

**Fig. 7:**
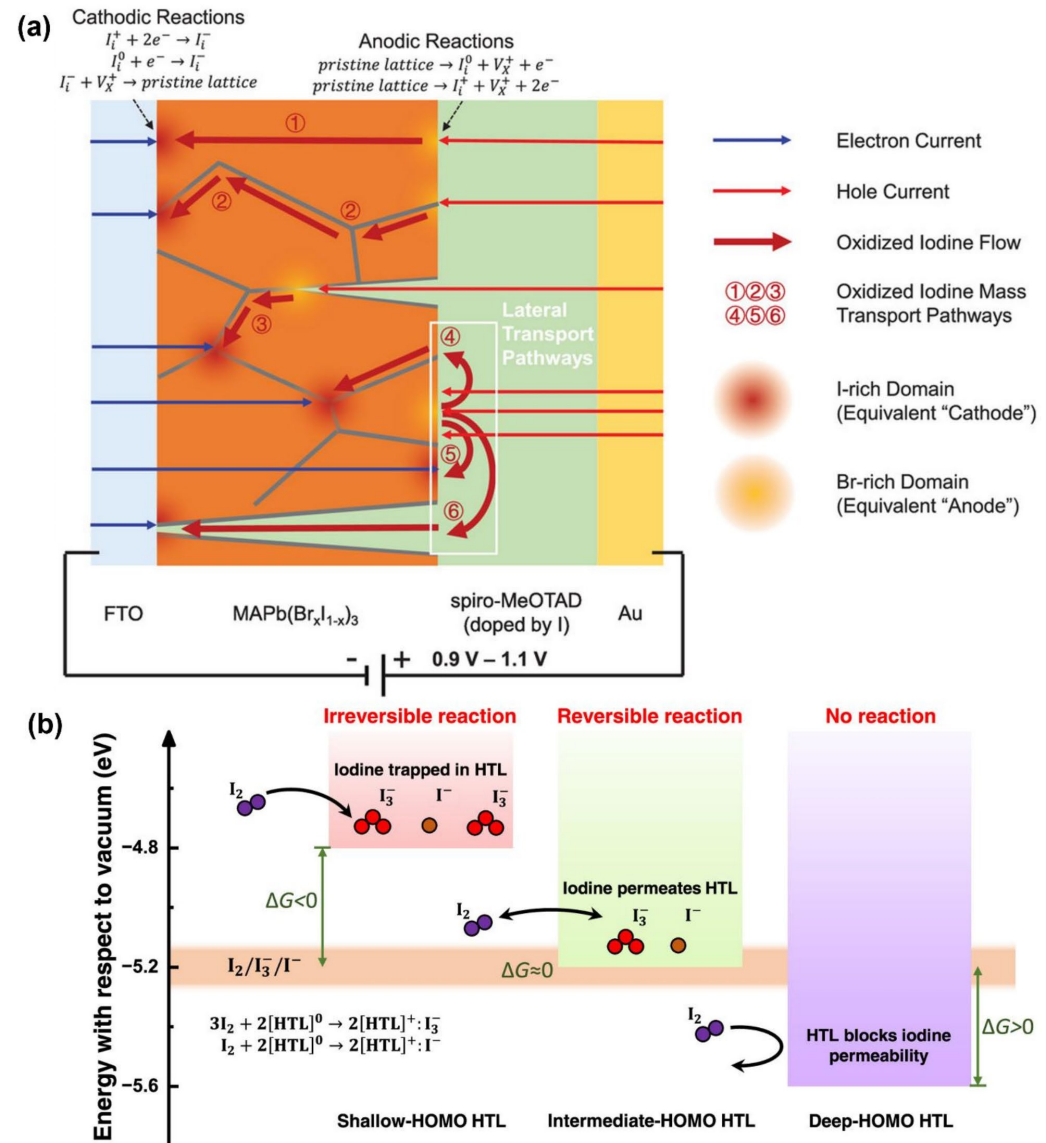
(**a**) Schematic illustration of oxidized iodine transport pathway in an FTO/MAPb (Br_*x*_I_1−*x*_)/spiro-MeOTAD/Au structure under voltage bias. Reproduced with permission [106]. Copyright 2022, Wiley. (**b**) Schematic of interaction between I_2_ and HTL with different HOMO levels. Reproduced with permission [81]. Copyright 2023, American Chemical Society

In mixed-halide (Br/I) perovskite devices, the applied high voltage biases can lead to voltage-induced halide segregation, which can impact the halide phase stability and bandgap tunability in the mixed halide perovskite devices [101–105]. Importantly, the influence of $\:{I}_{X}^{-}/{I}_{i}^{+}$ redox shuttles on halide segregation under applied bias (forward and reverse) is crucial for determining the stability of the mixed halides [40, 106, 107]. The lower oxidation potential of iodide compared to bromide leads to iodide oxidation earlier. Each redox shuttle reaction of iodide includes the oxidation of iodide at the anode (Eqs. 3–4) and reduction at the cathode (Eqs. 5–7), respectively (Fig. 7a) [108].

3 $$\:{I}_{X}^{-}\to\:{I}_{i}^{0}+{V}_{X}^{+}+\:{e}^{-}$$

4 $$\:{I}_{X}^{-}\to\:{I}_{i}^{+}+{V}_{X}^{+}+\:2{e}^{-}$$

5 $$\:{I}_{i}^{+}+\:2{e}^{-}\to\:{I}_{i}^{-}$$

6 $$\:{I}_{i}^{0}+\:{e}^{-}\to\:{I}_{i}^{-}$$

7 $$\:{I}_{i}^{-}+{V}_{X}^{+}\to\:{I}_{X}^{-}$$

Within a pristine lattice, iodide ions ($\:{I}_{X}^{-})$ undergo oxidation at the anode to form interstitial iodine cations $\:{(I}_{i}^{+})$ with iodide vacancy (V_x_^+^) (Eqs. 3–4). These iodine cations diffuse across crystal lattice and accumulate near the cathode (negative electrode), creating an iodide-rich domain (Eq. 5). Then, the oxidized iodine species are either reduced (Eq. 6) or re-incorporated into the lattice (Eq. 7) upon reduction reaction [40, 108]. Such iodide redox reaction makes halide ions mobile in the perovskite lattices.

This iodide oxidation upon photoelectrochemical hole injections leads to three distinct iodine transport scenarios depending on the energy level of the highest occupied molecular orbital (HOMO) of the HTL. Mass transport of oxidized iodine species across the grain boundary of perovskites and corresponding cathodic and anodic reactions is illustrated in Fig. 7a (pathways 1‒6) [109–112]. The interaction between oxidized iodine species (e.g., iodide/triiodide/iodine) and the HTL in the device is demonstrated in Fig. 7b [113, 114]. The energy level difference between the ionization energy (IE) of the HOMO of HTL and the oxidation potential of iodide can be defined as Gibbs free energy (Δ*G*), acting as a thermodynamic driving force for iodine diffusion permeability. Firstly, when the IE of the HOMO of the HTL is lower than the redox potential of I₂ (i.e., Δ*G* < 0), oxidized iodine species are rapidly permeable and trapped within the HTL. This process is thermodynamically favorable and irreversible [81, 114, 115]. Electrons from the HTL react irreversibly with the permeated iodine, generating thermodynamically stable products such as [HTL]^+^/I_3_^–^ or [HTL]^+^/I^–^ (Fig. 7b, left). Secondly, with an intermediate-HOMO HTL, where the thermodynamic driving force is close to zero (i.e., Δ*G* ≈ 0), reversible electron transfer can occur between iodine and the HTL’s intermediate HOMO level, enabling a reversible reaction (Fig. 7b, middle). Thirdly, with a deep-HOMO HTL (Δ*G* > 0), the high thermodynamic barrier effectively prevents I_2_ permeation into the HTL, thereby suppressing any redox reactions and permeation of iodine species into the HTL (Fig. 7b, right). The HOMO energy level thus determines the stability of the mixed halide perovskites, and one can understand the thermodynamics and kinetics of these three types of iodide diffusion scenarios upon contact with different organic HTLs [81].

### Electrochemical bias-induced reversible and irreversible reactions

**Fig. 8:**
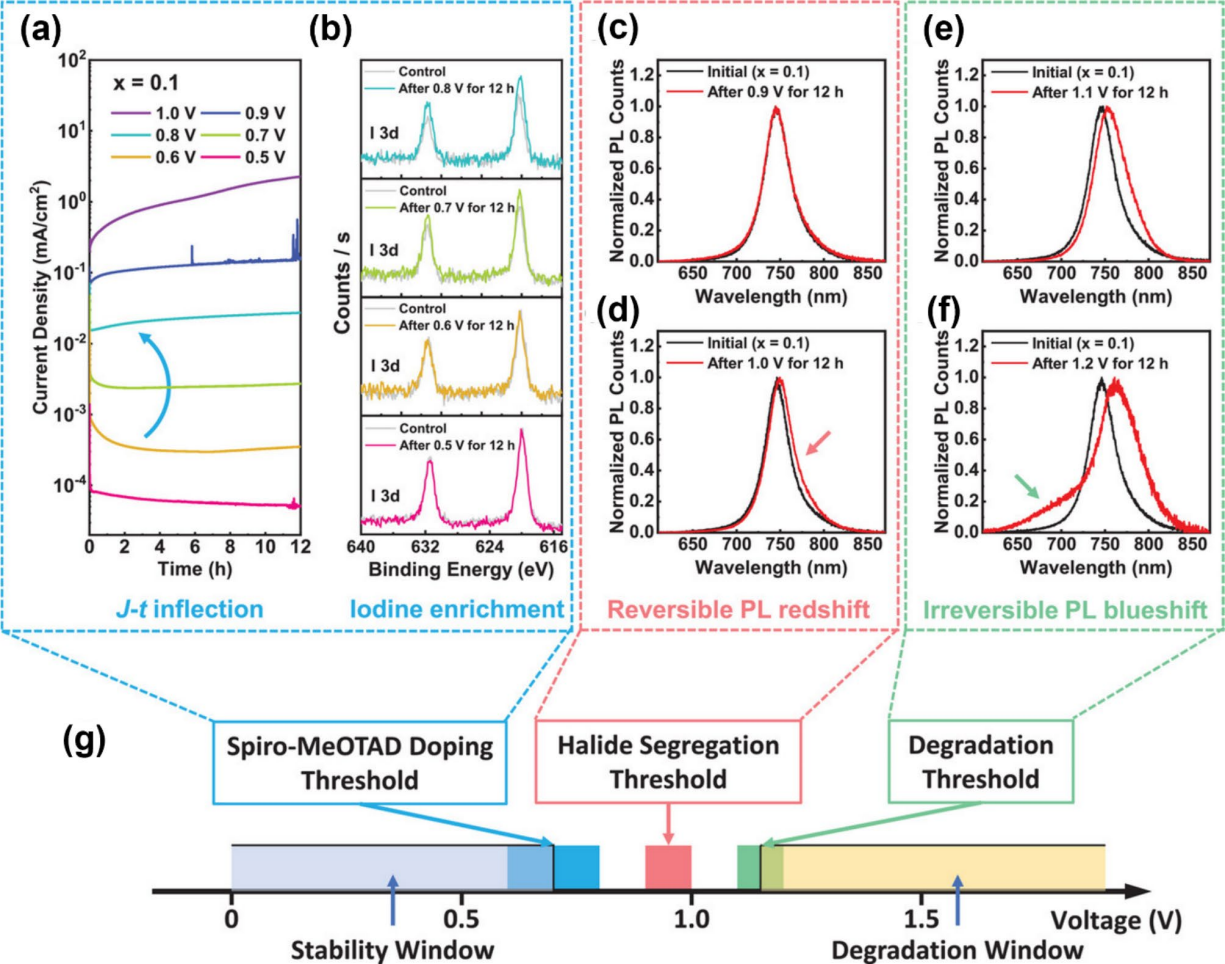
The MAPb(Br_0.1_I_0.9_)_3_ device used for determining the voltage threshold of spiro-MeOTAD doping: (**a**) current density measured under the voltage in the range of 0.5–1.0 V for 12 h (*J*-*t* curve) and (**b**) I_3d_ XPS spectra of unbiased as control and biased at 0.5–0.8 V for 12 h after the device was peeled off the top Au electrode. PL spectra before and after biasing for determining the voltage threshold for halide segregation at (**c**) 0.9 V and (**d**) 1.0 V, and for degradation at (**e**) 1.1 V and (**f**) 1.2 V for 12 h. (**g**) Schematic diagram for explanation of three voltage threshold observed in the device fabricated with an FTO/ MAPb(Br_0.1_I_0.9_)_3_/spiro-MeOTAD/Au. (**a-g**) Reproduced with permission [106]. Copyright 2022, Wiley

Mixed halide perovskites with a halide composition of I_0.8_Br_0.2_ (or lower bromide) of optimal bandgap (1.7 eV) are frequently employed as the light-harvesting layer in photovoltaic devices [116, 117]. These mixed halide perovskites, however, experience a series of sequential processes with increasing bias potential: halide doping into the spiro-MeOTAD layer (at 0.7 V), halide segregation (at 0.9–1.0 V), and halide expulsion and overall degradation (at 1.1–1.2 V), respectively (Fig. 8g) [118–120]. Therefore, the thermodynamic stability window for each of these processes needs to be defined through spectroelectrochemical experiments [106].

Rand and coworkers employed a perovskite device architecture consisting of FTO/MAPb(Br_0.1_I_0.9_)/2,2’,7,7’-tetrakis(*N*,*N*-di-*p*-methoxyphenylamino)-9,9’-spirobifluorene (spiro-MeOTAD)/Au [121, 122] to examine voltage window for inducing reversible or irreversible changes in the mixed halides. When being subjected to either voltage bias or light illumination, current density‒time (*J*‒*t*) profiles were recorded to understand the light- and voltage-driven halide ion migration and corresponding halide doping into HTL [83, 101, 107, 123, 124]. Initially, increasing bias voltage up to 0.8 V (for 12 h) led to an increase in current density over time (Fig. 8a). The increased current density at elevated bias voltage was associated with the diffusion of iodine/triiodide/iodide species into the spiro-MeOTAD HTL layer. X-ray photoelectron spectroscopy (XPS) measurements further demonstrated an increase in the I_3d_ peak area of spiro-MeOTAD HTL after biasing the film at 0.7–0.8 V for 12 h compared to the control sample (unbiased) (Fig. 8b). The higher HOMO energy level of spiro-MeOTAD relative to the iodide oxidation potential created a thermodynamically favorable environment for irreversible halide doping into the HTL (Fig. 7b, left). Combined spectroelectrochemical measurement (*J*‒*t* profile) in conjunction with XPS compositional analysis, iodide doping thresholds voltage could be determined to be 0.7 ± 0.1 V (for *x* = 0) and 1.1 ± 0.1 V (for *x* = 1), respectively [106]. Again, the reversibility of the iodide doping reaction into the HTL is governed by thermodynamic driving forces (Δ*G*), which correspond to the energy difference between the HOMO level of the HTL and the iodide oxidation potential.

Halide segregation, a reversible process occurring below the irreversible degradation threshold voltage, can be similarly ascertained (Fig. 8g). McGehee and coworkers first reported that photoinduced halide segregation occurs in the mixed bromide-iodide halide perovskites with a higher concentration of bromide (> 20%) under photoirradiation. However, spectroelectrochemical measurements allowed for observing the halide segregation even at lower bromide composition of MAPb(Br_0.1_I_0.9_)_3_ which is known as photostable. By probing the appearance of the red-shifted PL spectrum in the mixed halide perovskites at the elevated voltage, one can determine the halide segregation threshold voltage [125–127]. Voltage-induced halide segregation of MAPb(Br_0.1_I_0.9_)_3_ occurs above 0.95 ± 0.05 V (Fig. 8c, d). While previous studies have reported photo-induced segregation only in MAPb(Br_*x*_I_1−*x*_)_3_ with *x* > 0.2 [124], this work highlights the possibility of halide segregation even with a lower *x* value of 0.1 (e.g., MAPb(Br_0.1_I_0.9_)_3_). Unless the applied voltage exceeds a degradation threshold voltage (i.e., irreversible transformation), the segregated halide can be reversibly restored to its original mixed halide composition under dark conditions.

The irreversible halide degradation threshold was observed by monitoring the PL spectra as well. Further increasing the bias resulted in the appearance of blue-shifted PL spectra in the MAPb(Br_0.1_I_0.9_)_3_ device above the threshold voltage of 1.1 V and 1.2 V (Fig. 8e). Such spectral change was a hallmark of halide degradation in the mixed halide perovskite devices. While red-shifted PL spectra within the halide segregation threshold recovered after 24 h in the dark (reversible), the blue-shifted PL spectra observed under voltage bias between 1.1 V and 1.2 V remained unchanged even after 60 h (irreversible). These irreversible changes in the PL spectra suggest the formation of isolated halides and irreversible chemical transformation. Spectroelectrochemical measurements can thus capture both reversible and irreversible structural changes reflected in PL emission spectra and are a powerful tool for determining such thermodynamic window of stable halide phase [106, 107].

### Electrochemically driven irreversible halide expulsion in mixed-halide perovskites

**Fig. 9:**
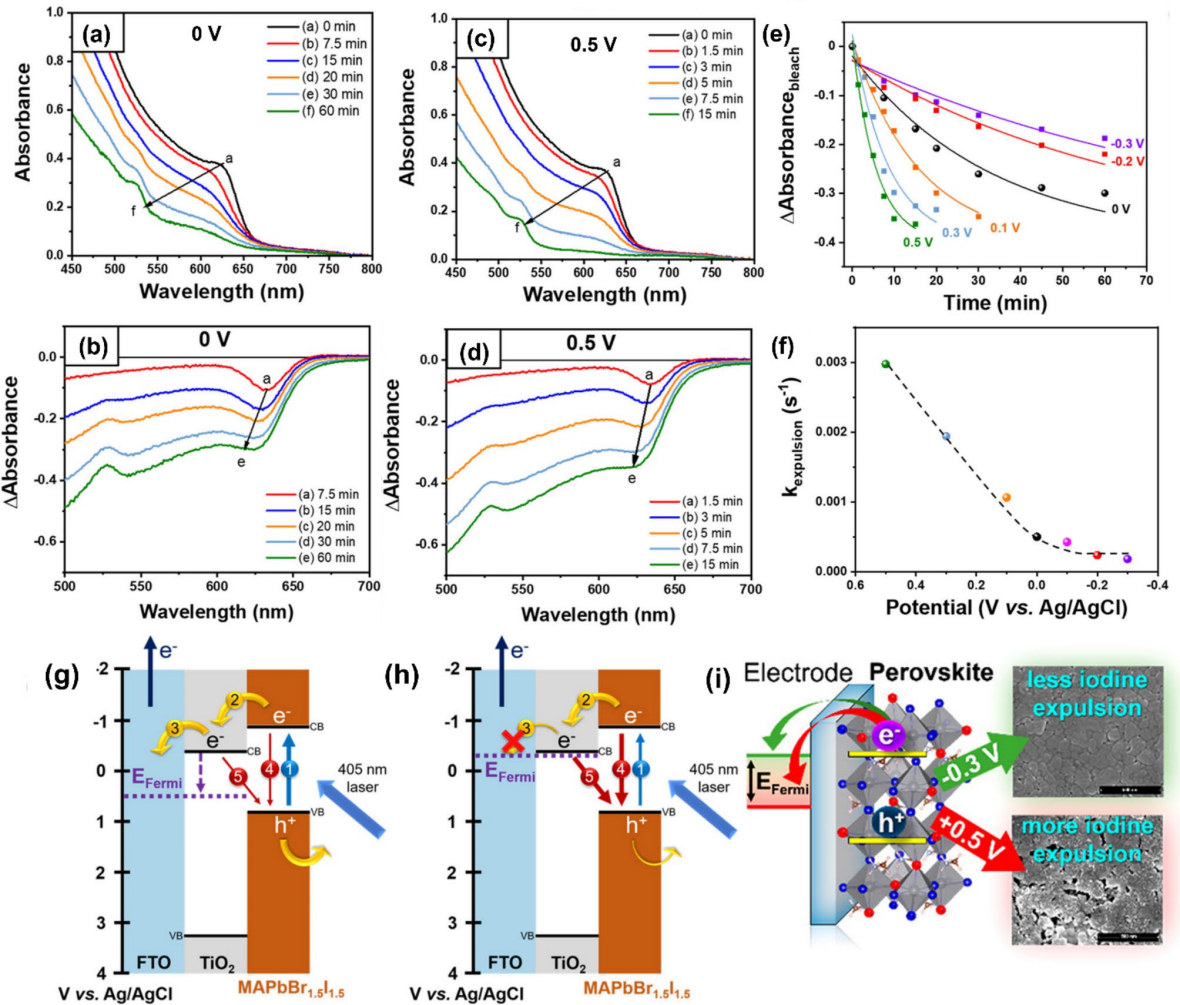
UV-vis absorption spectra of FTO/TiO_2_/MAPbBr_1.5_I_1.5_ films biased at (**a**) 0 V and (**c**) 0.5 V during 405 nm CW photoirradiation (50 mW cm^‒2^). (**b**,** d**) Difference absorption spectra of (**a**) and (**c**) to visualize band-edge absorption change during measurement. (**e**) Kinetic traces and monoexponential fits of 625 nm (bleach feature) for photoirradiated FTO/TiO_2_/MAPbBr_1.5_I_1.5_ films under various voltages (‒0.3 ~ 0.5 V). (**f**) The rate constant (*k*_explusion_) of iodine expulsion, depending on potential (V vs. Ag/AgCl), determined by the kinetic analysis presented in panel **e**. Schematic diagram of the pathway for charge carriers within the band structure of FTO/TiO_2_/MAPbBr_1.5_I_1.5_ films photoirradiated at (**g**) positive bias (+ 0.5 V) and (**h**) negative bias (‒0.3 V vs. Ag/AgCl). (**i**) Illustration of electrochemically modulated *E*_Fermi_ in perovskite film and SEM images of perovskite films after applying bias and photoirradiation. (**a-i**) Reproduced with permission [52]. Copyright 2021, American Chemical Society

Since all optoelectronic devices function under an applied voltage bias (both positive and negative), deconvoluting the combined interplay of light and voltage stimulation on halide stability is crucial for improving the stability of halide perovskite devices [52, 81, 97, 99, 107, 114, 120]. DuBose et al. investigated the influence of such complex interplay between light and voltage on the mixed halide MAPbBr_1.5_I_1.5_ films deposited on an FTO/TiO_2_ substrate upon photoirradiation with a continuous-wave diode laser (405 nm, 50 mW cm^‒2^) with controlled electrochemical bias vs. Ag/AgCl (at positive, zero, and negative bias, respectively). The film was in contact with an electrolyte in solvent (TBAFP_6_ and DCM). The mixed-halide perovskite film exhibited iodide expulsion even at 0 V upon photoexciation for 60 min as evidenced by spectral changes seen in the absorption and difference absorption spectra (Fig. 9a, b). These changes were manifested by a concurrent blue shift and decrease (bleaching) of the excitonic peak of MAPbBr_1.5_I_1.5_ at 625 nm. Mixed halides were initially segregated and iodines were expelled from the lattices consequently.

The perovskite film under negative bias (‒0.3 V) exhibited minimal spectral change over 60 min, suggesting an incomplete iodine expulsion process. On the contrary, at a positive bias of + 0.5 V, iodine expulsion was expedited and completed within 15 min (Fig. 9c). The difference absorption spectrum (Fig. 9d) more clearly visualizes the changes in the band-edge absorption peak during iodine migration/expulsion at unbiased and + 0.5 V conditions. The kinetics of iodine expulsion (*k*_expulsion_), obtained by monoexponential fitting of the differential absorption peaks at the 625 nm bleach feature (Fig. 9e), indicates that *k*_expulsion_ values increased one order of magnitude from 0.00018 s to 1 (at ‒0.3 V) to 0.003 s^‒1^ (at + 0.5 V), respectively (Fig. 9f). External bias can control shift of electrode’s Fermi level (*E*_Fermi_), interfacial charge transfer from electrode and perovskite, and iodine migration and segregation kinetics as shown in the band diagram (Fig. 9g, h). When a positive bias (e.g., + 0.1 ~ 0.5 V) was applied, the *E*_Fermi_ shifted away from the conduction band of TiO_2_ (Fig. 9g), facilitating electron transfer from the mixed halide perovskite to TiO_2_ and subsequently to FTO. However, the remaining holes became trapped at iodide sites within the perovskite, leading to lattice instability. The accumulation of holes was facilitated by higher positive biases, leading to a faster iodine expulsion. In contrast, under negative bias (e.g., 0 ~ ‒0.3 V), the *E*_Fermi_ approached the conduction band of TiO_2_, favoring electron-hole recombination along pathways 4 and 5 in Fig. 9h. Depending on the Fermi level engineering of the electrode, corresponding charge carrier dynamics in the mixed halides was controlled, affecting the stability of the mixed halides under photoelectrochemical bias (Fig. 9i). This study highlights the potential for manipulating charge carrier behavior and iodine expulsion in mixed halide through external bias control.

### Reactivity and effect of a metal electrode on halide stability

**Fig. 10:**
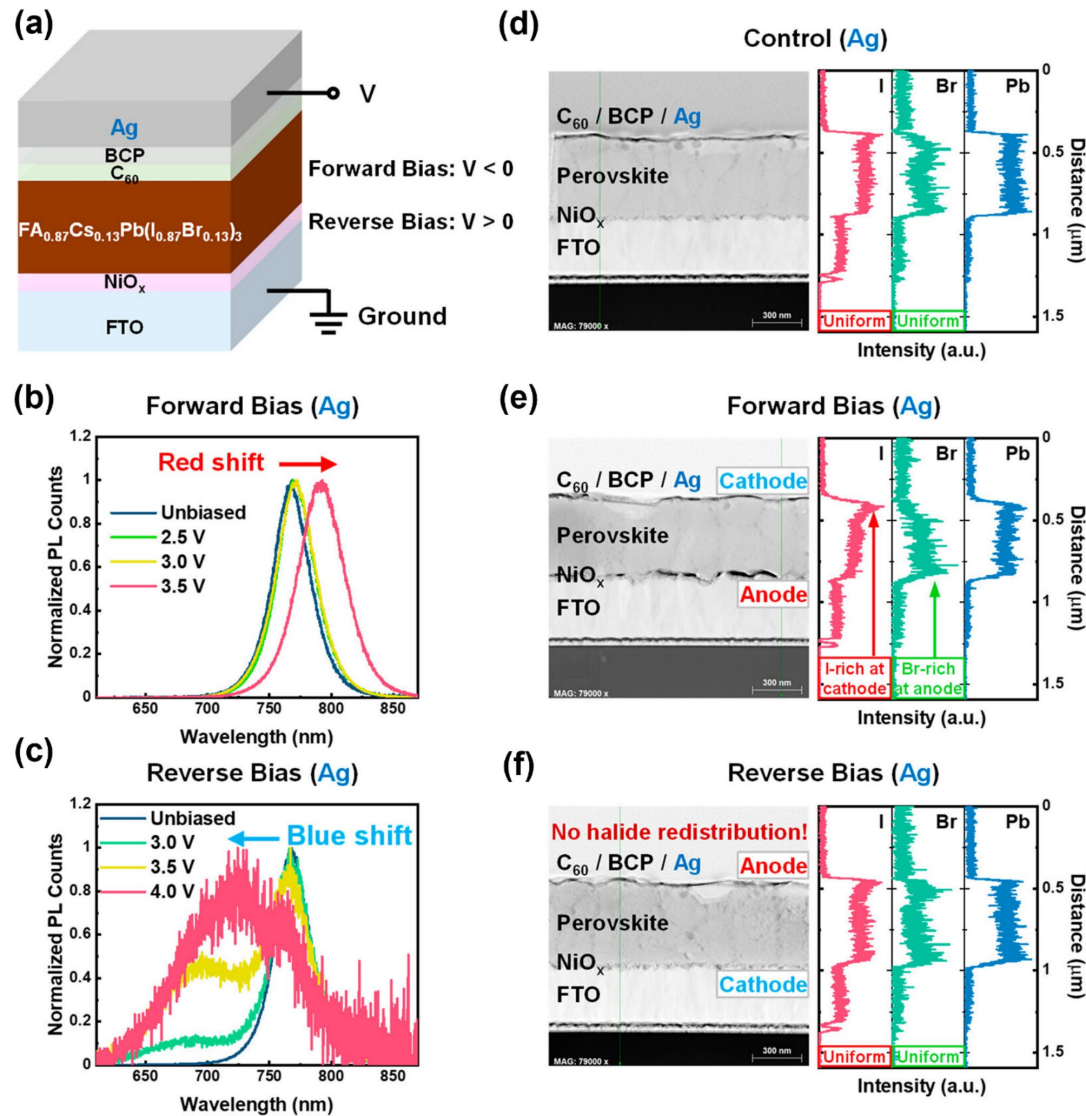
(**a**) Schematic illustration of the device for long-term voltage fabricated with FTO/NiO_x_/FA_0.87_Cs_0.13_Pb(I_0.87_Br_0.13_)_3_/C_60_/BCP/Ag. Normalized PL spectra of Ag device unbiased and after 12 h biasing at (**b**) forward bias (2.5, 3.0, and 3.5 V) and (**c**) reverse bias (3.0, 3.5, and 4.0 V). Cross-sectional high-angle annular dark field (HAADF) STEM images and EDX elemental distribution of Ag device under (**d**) unbiased, (**e**) forward bias, and (**f**) reverse bias. (**a-f**) Reproduced with permission [107]. Copyright 2023, American Chemical Society

**Fig. 11:**
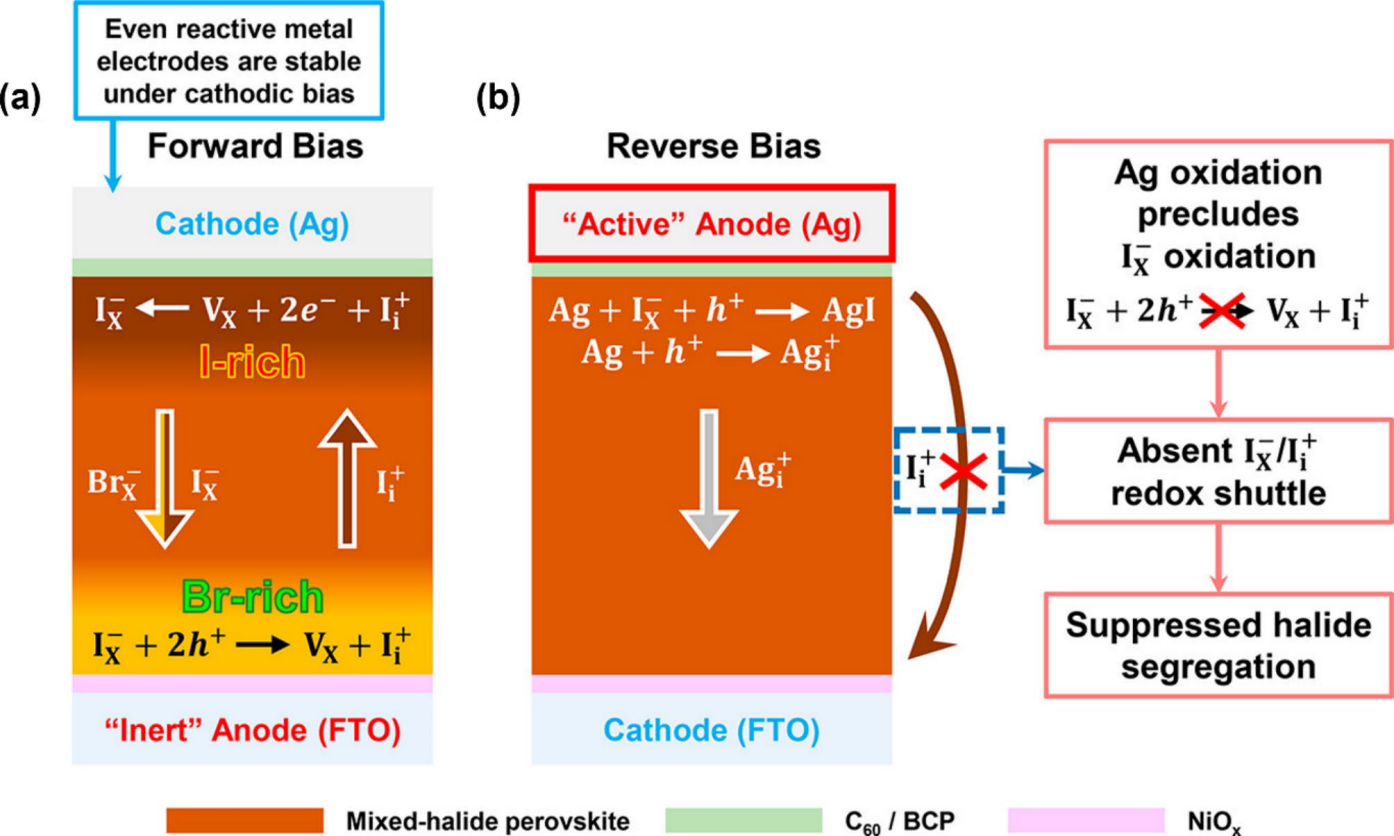
Schematic diagram explaining electrochemical reaction in the device with the redox-active anode (Ag) during (**a**) halide migration (segregation) process under forward bias and (**b**) suppressed halide migration (segregation) process that initiates step of Ag oxidation instead of halide oxidation. (**a-b**) Reproduced with permission [107]. Copyright 2023, American Chemical Society

Rand and coworkers also investigated the effect of metal electrodes on halide stability by comparing Au and Ag in perovskite devices. The proposed model involved C_60_/BCP/Ag perovskite solar cell architecture wherein Ag was used as an anode instead (Fig. 10a, d). Under forward bias, as expected in both Au and Ag devices where FTO/NiO_*x*_ was employed as the anode, halide segregation occurred, which was confirmed by the red-shifted PL spectra (Fig. 10b, e) [121, 124]. Scanning transmission electron microscopy coupled with energy-dispersive X-ray spectroscopy (STEM-EDX) analysis demonstrated the distinctive halide redistribution, generating the I-rich at the cathode and Br-rich at the anode, respectively (Fig. 10e). Halide segregation mechanism is demonstrated in Fig. 11a in more detail. Oxidation of iodides (I_*x*_^‒^) at the FTO/NiO_*x*_ anode interface created oxidized iodine and iodide vacancy. These mobile species are permeable through perovskite lattices toward the cathode wherein oxidized iodine is electrochemically reduced and returned to the lattice $\:{I}_{X}^{-}$ anions. Simultaneously, the halide vacancies near the anode are filled by $\:{I}_{X}^{-}$ and $\:{Br}_{X}^{-}$ anions that drift through the bulk mixed halide away from the cathode. Due to the difference in redox potential between bromide and iodide, only iodine species (more easily oxidized than bromide) can move back to the cathode in the form of oxidized I_i_^+^ across halide perovskite lattices. The oxidization potential of bromide species at the anode was much greater, suggesting a thermodynamically unfavorable bromide oxidation reaction. Consequently, the iodide and bromide were segregated and redistributed near the cathode and anode, respectively. The halide segregation and redistribution process across the Br/I mixed halide phases in the devices depended on the $\:{I}_{X}^{-}$/$\:{I}_{i}^{+}$ redox shuttle under different voltage biases with different polarities (forward and reverse bias) [107].

In the case of the Ag device with reverse bias, however, the suppressed halide segregation was observed in the PL spectrum and STEM-EDX measurements (Fig. 10c, f). No halide redistribution occurred in the Ag device under reverse bias [124]. Instead, the blue-shifted PL spectrum was seen, demonstrating the electrochemical degradation of the perovskite layer while maintaining uniform halide distribution revealed by STEM-EDX measurements (Fig. 10c, f). The underlying mechanism of the suppressed halide segregation in Ag devices under reverse bias is demonstrated in Fig. 11b. The active Ag anode was involved in prioritizing its own oxidation reaction over the oxidation of iodide species ($\:{I}_{X}^{-})$ according to the following reactions (Eq. 8):

8 $$\:Ag+{I}_{X}^{-}+{h}^{+}\:\to\:AgI$$

The absence of $\:{I}_{X}^{-}$ oxidation, which is crucial for the $\:{I}_{i}^{+}/{I}_{X}^{-}$ redox shuttle and halide segregation, prevented halide ion migration and segregation in mixed halide devices with the active Ag anode. Sacrificial oxidation of the active Ag electrode itself can aid in improving the stability of the mixed-halide perovskites. Overall, a comprehensive understanding of the interplay between the oxidation potentials of halide ions, metal electrodes, and layer interfaces is essential. The redox potential of the metal electrode significantly influences the stability of mixed halide perovskites, determining whether phase segregation occurs.

### Stability under the practical operational condition of cells

Numerous literature reports have explored the issue of halide ion migration, segregation, and subsequent degradation in halide perovskite materials. A primary cause of these phenomena is the formation of iodide vacancies (or more generally, halide defects) induced by iodide oxidation, which can facilitate halide ion diffusion. To mitigate or prevent halide ion migration and its associated problems, strategies such as passivating defect states with Lewis base ligands (including 2D molecules, alkyl or aromatic amines, phosphines, phosphine oxides, phosphonic acids, sulfonic acids, and polymers) and employing lower-dimensional perovskites with intercalated spacer ligands to increase the activation barrier for migration have been proposed [128]. From a practical standpoint, a crucial application of spectroelectrochemistry lies in assessing the stability of halide perovskites when integrated into devices (e.g., perovskite solar cells and perovskite light-emitting diodes). Under realistic operating conditions, absorption and emission spectra can be measured under open-circuit conditions or during device operation.

**Fig. 12:**
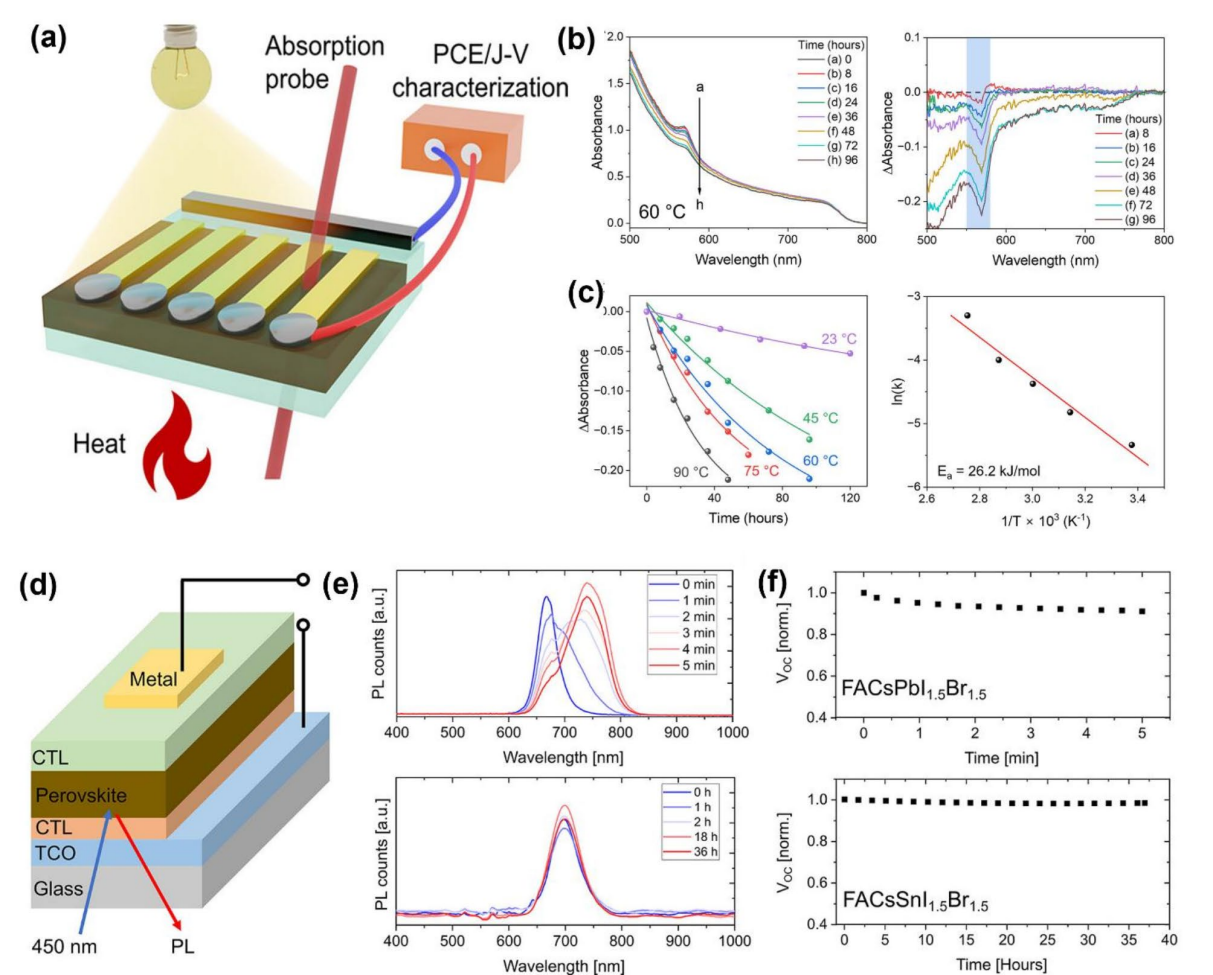
(**a**) Schematic illustration of a 2D/3D perovskite solar cell under heat and light irradiation. (**b**) Changes in absorption and difference absorption of the 2D/3D perovskite solar cell recorded at 60 °C. (**c**) Absorption intensity changes of the 2D/3D perovskite solar cell by monitoring 570 nm (corresponding to *n* = 2) at different temperatures (23–90 °C). (**a-c**) Reproduced with permission [129]. Copyright 2023, American Chemical Society. (**d**) Schematic illustration of mixed halide (Br: I = 50:50) perovskite solar cells. (**e**) In-situ tracking of PL emission for the perovskite solar cell at open circuit condition (top: FA_0.87_Cs_0.13_PbI_1.5_Br_1.5_, bottom: FA_0.83_Cs_0.17_SnI_1.5_Br_1.5_) under a 450 nm laser diode with a power of 50 mW cm^‒2^. (**f**) Tracking V_oc_ of the mixed halide Pb-based (top) and Sn-based (bottom) perovskite solar cells over time. (**d-f**) Reproduced with permission [130]. Copyright 2023, American Chemical Society

Figure 12a illustrates the experimental setup designed to characterize two-/three-dimensional (2D/3D) perovskite solar cells under operational conditions. By monitoring changes in absorption spectra, one can gain a deeper understanding of the interfacial stability of 2D/3D BA_2_MAPb_2_I_7_/MAPbI_3_ perovskites. Upon heating the 2D/3D perovskite film to 60 °C and tracking absorption changes, a decrease in band-edge absorption at 570 nm (corresponding to *n* = 2 perovskites) was observed over time (Fig. 12b). This suggests that 2D butylammonium (BA) molecules destabilize under elevated temperatures, leading to chemical interactions with 3D perovskites and the formation of a gradient 2D/3D phase under operational conditions. By analyzing the difference absorption spectra and monitoring peak intensity changes at 570 nm, the Kamat group determined the kinetic rate constant (*k*) at various temperatures (Fig. 12b). An Arrhenius relationship between ln *k* and 1/T was established, yielding an activation energy of 26.2 kJ mol^‒1^ (Fig. 12c). These findings indicate that the 2D/3D perovskite interface is relatively stable at room temperature but undergoes rapid destabilization at elevated temperatures due to interlayer mixing between 2D and 3D phases. This study provides crucial insights into the stability of 2D/3D perovskite solar cells, revealing that they do not always exhibit superior performance and stability compared to 3D perovskite counterparts [129].

Similarly, in-situ PL emission tracking of perovskite solar cells can reveal insights into their operational stability. Figure 12d illustrates the experimental setup for investigating the photoirradiation of a mixed halide perovskite film (Br: I = 50:50) in a solar cell device using a continuous wave 450 nm laser diode excitation. Under open-circuit conditions, by monitoring the PL emission from the perovskite layer over time, it becomes evident that Sn-based perovskites exhibit superior mixed halide phase stability compared to their Pb-based counterparts, despite having similar halide and A-site compositions (Fig. 12e). Furthermore, by recording the open-circuit voltage (V_oc_) changes induced by 450 nm laser diode excitation, it is observed that Sn-based perovskites demonstrate less V_oc_ degradation under photoirradiation than Pb-based perovskites (Fig. 12f). The enhanced stability of the Sn-based perovskites can be attributed to the increased activation barrier for halide migration, resulting from stronger Sn‒I bond compared to Pb‒I bond. This study, therefore, establishes a correlation between halide stability and PL emission spectra changes, as well as solar cell performance and stability and V_oc_ variations [130].

## Future outlooks and perspectives

Halide perovskites have emerged as highly promising next-generation optoelectronic materials; however, their practical application is significantly hindered by stability concerns. The intrinsic ionic nature of these materials, characterized by soft crystalline lattices, renders them highly susceptible to external stimuli such as light and electric fields. This susceptibility often manifests in halide ion migration-induced instability. A fundamental understanding of halide oxidation processes under illumination and electric bias is therefore crucial for developing strategies to enhance stability. By integrating thermodynamic and kinetic considerations, spectroelectrochemical techniques have been employed to simulate the operational conditions of halide perovskite devices, such as photovoltaic cells and light-emitting diodes. These methods enable the investigation of spectral changes induced by charge injection under controlled light and electric field conditions.

Spectroelectrochemical analysis has revealed that iodide oxidation plays a critical role in determining the stability of both pure and mixed-halide perovskites. Due to the lower oxidation potential of iodide compared to chloride and bromide, initial iodide oxidation occurs. This process leads to the formation of iodide vacancies, which act as pathways for halide ion migration (or defect transport) across the halide sublattices. The oxidized iodide species (I_i_^+^) migrating into the interstitials are significantly more mobile than the reduced, lattice-bound iodide (I_X_). This enhanced mobility leads to several consequences including (i) further halide ion doping into the HTL (e.g., spiro-MeOTAD), (ii) interaction with the electrode, forming metal iodides, (iii) halide segregation in the case of mixed-halide perovskites (reversible: once the halide is within the perovskite lattice), and (iv) irreversible degradation or expulsion of halides from the lattice (shifting the iodide chemical equilibrium).

To mitigate such problems mainly arising from iodide oxidation, preventing the oxidation process of I^‒^/I_i_^+^, mass transfer of iodide vacancy, and flux (through which halide migration occurs) need to be considered. It is challenging to prevent intrinsic iodide oxidation due mainly to the thermodynamically favorable redox potential of I^‒^/I^+^ or I^‒^/I_3_^‒^ under photochemical and electrochemical excitation. Therefore, the selection of charge transport layers and electrodes with appropriate band alignments plays a vital role. By blocking the migration (permeability) of oxidized iodine in the redox shuttling process of iodine species and by tuning the HOMO energy level of HTL (thermodynamic control over iodide permeability) or Fermi energy of electrode (Ag vs. Au), it should be possible to improve the stability. It is important to recognize that any process involving iodide oxidation and migration can destabilize the devices, impacting both performance and longevity. Therefore, understanding the mechanisms linking photoelectrochemically driven iodide oxidation and vacancy-mediated iodide migration under operation becomes a critical factor in stabilizing halide perovskite devices.

Moving forward, one can consider thermodynamic and/or kinetic control over halide migration in halide perovskites. From the thermodynamic pathway, engineering band alignment between the contacting charge transport layer (or electrode) and halide perovskites can mitigate halide migration. From the kinetic perspective, control over the iodide migration barrier by introducing lower dimensional perovskites with increased bandgap and passivating the halide vacancies with surface-capping ligands or introduction of lower dimensional perovskites (e.g., 2D phase) on 3D perovskites is beneficial in suppressing the iodide migration. The 2D molecules or ligands can passivate the surface defects, suppress halide vacancy formation, and ultimately hinder iodide migration across the anion sublattice.

## Data Availability

Not applicable.
